# Digital health technology to support patient-centered shared decision making at point of care for juvenile idiopathic arthritis

**DOI:** 10.3389/fped.2024.1457538

**Published:** 2024-10-25

**Authors:** Bin Huang, Michal Kouril, Chen Chen, Nancy M. Daraiseh, Kerry Ferraro, Melissa L. Mannion, Hermine I. Brunner, Daniel J. Lovell, Esi M. Morgan

**Affiliations:** ^1^ Division of Biostatistics and Epidemiology, Cincinnati Children’s Hospital Medical Center, Cincinnati, OH, United States; ^2^ Department of Pediatrics, College of Medicine, University of Cincinnati, Cincinnati, OH, United States; ^3^ Division of Biomedical Informatics, Cincinnati Children’s Hospital, Cincinnati, OH, United States; ^4^ JIA Parent, Lower Gwynedd, PA, United States; ^5^ Department of Pediatrics, University of Alabama at Birmingham, Birmingham, AL, United States; ^6^ Division of Rheumatology, Cincinnati Children’s Hospital Medical Center, Cincinnati, OH, United States; ^7^ Division of Rheumatology, Department of Pediatrics, Seattle Children’s Hospital, Seattle, WA, United States; ^8^ Department of Pediatrics, University of Washington School of Medicine, Seattle, WA, United States

**Keywords:** digital health technology, shared decision making, clinical decision support system, juvenile idiopathic arthritis, learning network model, registry analysis, personalized medicine

## Abstract

Despite availability of multiple FDA approved therapies, many children with juvenile idiopathic arthritis (JIA) suffer pain and disability due to uncontrolled disease. The term JIA includes a heterogeneous set of conditions unified by chronic inflammatory arthritis, collectively affecting 1:1,000 children. When reviewing treatment options with families the rheumatologist currently refers to the experience of the average patient in relatively small controlled clinical trials, to consensus-based treatment plans, or increasingly the choice is dictated by the formulary restrictions of insurance payers. The current paradigm for treatment selection does not incorporate real-world evidence of treatment effectiveness centered to the individual patients with whom decisions are to be made. Treatment decisions based on the evidence of the average patient are not optimized to reflect the unique clinical characteristics of an individual with JIA and their disease course, nor does it account for heterogeneous treatment effects. To guide treatment choices centered around each patient, we describe a novel concept of utilizing digital health technology to bring patient-centered information into shared decision-making discussions based on comparative effectiveness analysis of electronic health record or observational clinical registry data of patients with similar characteristics. The envisioned digital tool will organize and present data relevant to the individual patient and enable evidence-based individualized treatment decision making when used in a collaborative manner with the patient family and rheumatologist. Capabilities in digital health technology, data capturing, and analytical methodologies are ripe for this endeavor. This brings the concept of a learning health system directly to the point of care.

## Introduction

Juvenile Idiopathic Arthritis (JIA) is an umbrella term for heterogeneous chronic inflammatory arthritic conditions of childhood onset that neither have a known etiology nor a cure. An estimated 300,000 children have a rheumatologic condition, and an estimated 80,000 children in the United States have some form of JIA. Despite the availability of multiple FDA approved therapies, JIA is a condition that remains uncontrolled for many children who suffer negative health outcomes, including chronic pain, growth disturbances and functional disability. Of the seven subtypes, one of the most difficult to control is polyarticular JIA (pJIA), characterized as having five or more inflamed joints, with features like rheumatoid arthritis in adults (1, 2). In a setting of multiple available treatment options, only about 40% of pJIA patients achieve a controlled disease state (3).

The lack of satisfactory disease control is likely multifactorial, but one known important factor that we seek to address using digital technology is the heterogeneity of treatment responses. Patients with JIA may respond to the same treatment differently, perhaps due to differing biology, comorbidities, or genetic factors, but heterogeneity of outcomes may also be due to the timing of treatment with respect to diagnosis, disease prognosis, use of concomitant medication, treatment duration or treatment adherence. We anticipate better health outcomes could be attained with reliable identification, selection, and prescription of the optimal treatment for a given patient chosen from currently available candidate treatments by accounting for heterogeneous factors in a comparative effectiveness analyses model.

The ability to select optimal treatments at time of diagnosis with inflammatory arthritis is vital as there seems to be a window of opportunity wherein the early achievement of clinical inactive disease within one year of diagnosis is a strong predictor of better long-term clinical and health related quality of life outcomes (4, 5). Thus it is important to understand and account for the role of heterogeneity of treatment effects in selecting initial JIA treatment.

## Heterogeneity of treatment effects

Heterogeneity of treatment effect (HTE) refers to differential and non-random effects of treatments on individuals in a population compared to others, indicating that there are clinically relevant subgroups who may have different benefits (or lack thereof) compared to others (6). This is to be distinguished from the average treatment effect (ATE) estimated in studies, which would suggest that a treatment would have a similar effect across subgroups, or patients with heterogeneous characteristics (6). Historically, ATE can only be estimated from randomized controlled clinical trials (RCTs). Thanks to the theory and analytical development of statistical causal inference method, we now can utilize data observed from real clinical encounters to inform how different treatment approaches may compare. Current clinical decision-making in the main is based on ATE. There are multiple rationales for standardization of medical care and use of protocols to reduce variation in care, including reduced potential for medical error, decreased health inequities, and increased ability to perform comparative effectiveness studies in observational data. Therefore, clinicians, healthcare systems and researchers alike are motivated to pursue uniform treatment approaches across patients. However, treatment by protocols that do not consider prognostic factors and clinical presentation may not yield the best outcomes for individual patients, nor the population. Seeking consensus treatment plans that work for an “average” patient may not serve all patients due to heterogeneity of conditions and response. We believe a digital health technology (DHT) solution can be created to leverage comparative effectiveness analyses of relevant clinical patient information and present a data dashboard at point of care (POC) to inform a patient-centered and standardized care approach. We anticipate, with consideration of individual patient features such as subtype of JIA, duration of disease at diagnosis, serologic markers, sex, age, and response to prior treatments, and synthesizing the collective wisdom/experiences of care episodes, such a DHT could improve clinical outcomes. Variation in treatment across individuals informed by the DHT and based on HTE would be warranted.

## Data sources to inform treatment decision-making

RCTs have been the primary data source used to establish the efficacy of medical treatments. However, RCTs are often relatively small for rare conditions such as JIA, and thus are limited in generating robust information on HTEs. Innovative trial designs such as pragmatic clinical trials, randomized withdrawal trials, and sequential multi-stage adaptive randomized trial (SMART) have been pursued recently. Yet the averaged treatment effect remains to be the primary quantity of estimation, due to methodologic challenges related to estimating patient-centered treatment effect.

Increasingly, sophisticated bioinformatics technology captures rich clinical information reflecting clinical decisions that were made at the point-of-care (POC) and the information that factored into the decision. The establishment of multi-center learning health networks that implement common data models for clinical data entry into a shared registry make it possible to combine data from multiple centers on a clinic population with data reflecting real-world treatment practices and patient outcomes. Compared to RCT data, a learning health network (LHN) registry that seeks the complete population representation for the purposes of quality improvement (QI), may offer more generalizable data and robust evidence to inform treatment effectiveness for heterogeneous and dynamic conditions such as JIA.

## How to estimate patient-centered treatment effect

To estimate HTEs, the historical approach was to examine treatment by covariate interactions. However, this approach requires testing multiple interaction terms, which raises multiplicity issues. When not addressed, this may lead to inflated type I error (7). Furthermore, such an approach imposes strong modeling assumptions, e.g., linear regression, which could seriously bias the effect estimates when a model is mis-specified. In addition, it is not always clear what covariates may modify the treatment effect, and how the covariates interact with treatment and among themselves. The challenges of HTEs are further complicated, due to treatment-by-indication bias, information biases, missing and/or censored data. Even in the RCT setting, estimation of HTEs is often complicated by intercurrent events such as early termination, loss-to-follow up, treatment switching, and/or use of rescue medications.

Statistical causal inference methods addressing HTEs largely fall within two categories – subgroup finding and conditional averaged treatment effect (CATE). Subgroup finding searches among the feature space defined by preselected patient characteristics such as age, sex, and disease subtype, identifying the subgroups (often a combinations of multiple features) that present distinct treatment effects than the averaged effect. This can be used to derive clinical decision rules based on simple and commonly available patient features and obtain estimates of subgroup averaged treatment effect (SATE). The CATE on the other hand, estimates effect of treatment conditional on the values of feature space, often leveraging on the semiparametric or nonparametric modeling algorithms such as random forest. Bayesian adaptive regression tree (BART) modeling is widely recognized to provide well performed ATE and has been suggested to model CATE (8). A concern with a highly flexible modeling approach is overfitting, which could lead to overly confident estimates that are not reproducible in another study sample. Setting aside a subsample of data may help achieving better “honest” inferences to the estimated treatment effect using an adapted random forest approach, where the node splitting criteria is designed to optimally create multiple subgroups of HTEs (9). When treatment-by-indication confounding bias is of concern, doubly robust causal inference methods are used to introduce additional safeguards against potential model misspecification (10–13). Bayesian Gaussian Process (GP) utilizing GP covariance function as a matching tool, can provide a Bayesian's doubly robust approach (14, 15). The Bayesian approach is well-suited for synthesizing and updating knowledge for informing evidence-based decision making. The Bayesian framework, where the prior represents the existing knowledge, uses new data to update the prior and produce the posterior that represents the updated knowledge synthesizing both past and new learning. These Bayesian approaches can explicitly consider the multiplicity issue (16), search subgroups with distinct treatment effects (17), and be coupled with nonparametric models to mitigate the model misspecification issue in HTEs (18). The decision-based Bayesian causal inference method can be used to identify patients who may experience clinical meaningful improvements from a given treatment (17).

The causal inference methods HTEs brought us much closer to better understanding patient centered treatment effect. However, much work remains to rigorously validate the HTEs provision of causal inference at the individual level to inform individual treatment effect (ITE). For example, CATE and SATE neglect the inherent variability in response measurements or due to finite samples, yet consideration of these variabilities is critical to inform decision-making. Building on the existing HTE methods, we seek to identify a better performing approach with the goal of delivering relevant and valid comparative effectiveness treatment evidence for each individual patient. Towards this goal, the chosen method to inform treatment decisions at point-of-care should meet the following criteria: (a) it provides an accurate estimate of ITE; (b) it provides nominal level of confidence in a treatment choice; and (c) it is computationally efficient and feasible at point-of-care.

To validate methods for informing ITE, we need to assess the performance of the method with an independent sample of the “target” patient with whom the decisions are to be made. We may do so by taking a leave-one-out (LOO) approach with the existing data source. However, due to the inherent variabilities in responses and sample heterogeneity, the observed outcome for the out-of-sample “target” patient is only a random realization of many versions of possible outcomes. This imposes the seemly infeasible task of performing validation for ITE, unless we have access to the expert clinicians and consensus agreement. By having access to the data recorded from the real clinical encounters of thousands of patients cared for by hundreds of physicians, we in fact do have access to the requisite expert opinions. For each individual patient sitting in the doctor's office, we could identify the subsample of patients in the database that resemble or are “alike patients”. This means, the treatment decisions made and the outcomes following the corresponding decisions can be extracted. The summary statistic pooling data from all physicians could serve as an anchor point, allowing us to validate and compare the performance of different causal inference methods for informing patient centered decisions.

The top performing models can be implemented in a DHT, which will take the input of patient data in the EMR, clinical registries, and clinical trial data inputs. The DHT may also be updated with additional data information accumulated as more data is made available. The DHT should generate output that provides patient-centered estimate of treatment effect based on patient characteristics in a format designed in a manner to review and discuss with the patient.

## From digital health technology to shared decision making

Clinical decision support (CDS) is a model element to improving chronic illness care (19). In rheumatology, active monitoring of disease status and medication adjustment if treatment targets are not achieved—a strategy called “treat to target” (T2T)—results in tighter disease control (20), and better long-term outcomes. Adding a CDS tool increases the impact of T2T (21), and a study of T2T with CDS in JIA suggested that use of CDS over time could potentially ameliorate racial disparities in disease activity that had been identified at diagnosis (baseline) (22). However, in clinical practice, a T2T approach in JIA is limited by the lack of evidence-based, accessible at POC CDS on next best treatment decisions that would be expected to result in better disease control considering the patient characteristics.

**Figure 1:**
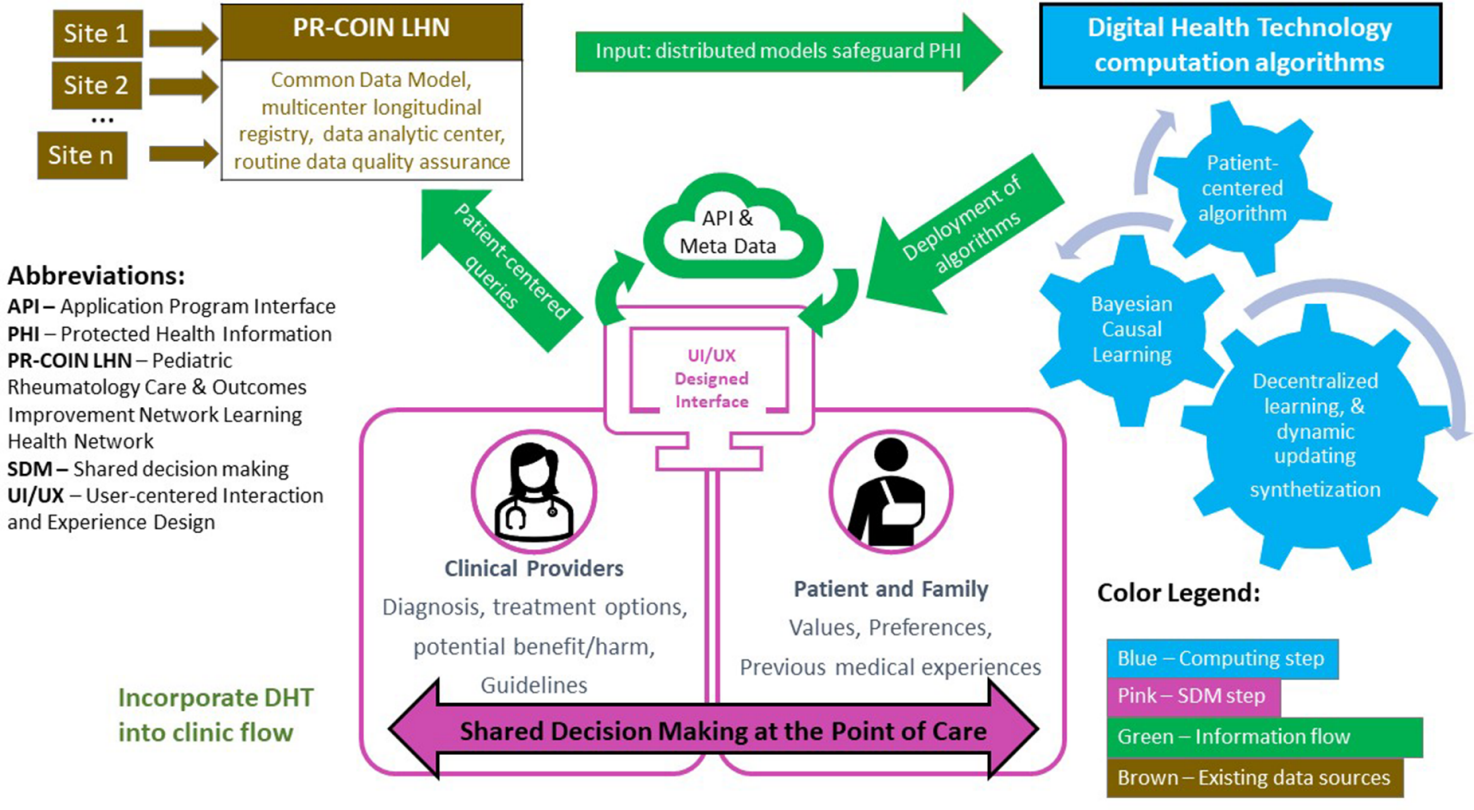
Conceptual framework: digital health technology enabled patient-centered shared decision making at point of care.

Recent consensus recommendations from pediatric bioethicists stated, “to respect children and promote their wellbeing, clinicians and parents should inform pediatric patients of salient information and invite their perspective to the degree that doing so is developmentally appropriate.” (23) To this end, the digital health tool that will be presented as a CDS to inform treatment decisions at POC should be designed with the intent to be both provider and patient facing to support collaborative shared decision making with patients and their care takers. The concept is illustrated in the Figure 1. The algorithm informing the digital health tool includes an age span of 1–18 years. Initial development and analysis of the tool centers on decision making with parents, but with a clinical goal of including patients in the process. Future research will study the dynamics of parent-child dyads in the decision process.

## Why a patient-centered shared-decision making tool can make a difference

Human intelligence learns from what we observe and applies learnings to future decisions. Prior to the big data age, clinicians learned from published textbooks or the medical literature, their past experiences, and from communications with their peers. For each future patient, the more we accumulate past knowledge relevant to the patient, the better we are at making treatment decisions. As a result of advances in immunology, together with biotechnology innovations and modern pharmaceutical product development, the medical field has made great advancement in treating JIA disease conditions with improved health outcomes. However, treatment of JIA, a heterogeneous group of conditions, is complex, and the disease course unpredictable. Even in this increasingly rich information environment JIA treatment continues to involve guesswork and anecdote, subject to human error, bias, and unwarranted variation in care.

What if we could add a patient-centered learning algorithm into decision making at POC? Powered by such an algorithm validated in a research setting (14), we aim to build an interactive, user-friendly CDS to support shared decision-making at POC. The use of such a tool can address health equity concerns by standardizing the evidence-based approach taken with each patient. Resulting treatment variation between patients would be warranted based on computational predictive analytics of most effective treatment resulting in individualized care. Rather than “one-size-fits-all”, the individualized treatment approach will consider, through algorithm learning, what has worked well in similar patients.

## Application to a learning health system: development and testing

Healthcare equity is a central consideration in the design and implementation of any new DHT. The Agency for Healthcare Research and Quality has developed a Digital Healthcare Equity Framework guided by principles that the new technology should reduce inequities, be person-centered, be inclusive in development, be able to be implemented in diverse settings, be cognizant of policy, and be focused on outcomes (24). The framework details aspects of development to consider developing a DHT that promotes health equity. These recommended developmental approaches include engagement of diverse potential end-users, identification of potential cultural barriers to use to design around, when developing workflows paying attention to access to information technology, obtaining iterative feedback on whether technology is serving needs of the end-users, and inclusion of representative data in development ^(^24). These guiding principles and domains are important to bear in mind with any healthcare delivery improvement. Usability surveys and quality measures (process, outcomes, healthcare experience) will be stratified by demographic features or social determinants of health throughout pilot testing of a new DHT and after implementation to monitor for equitable application.

A LHN is an optimal context to develop, test and deploy such a DHT. The Pediatric Rheumatology Care and Outcomes Improvement Network (PR-COIN) is such a LHN on a shared clinical registry populated by electronic data transfer from the EMR, local databases, or manual data entry extracted from the EMR (25). Participating sites are unified in a shared and relentless focus on improving outcomes of all JIA patients using QI methods, with attention to standardized care, avoidance and mitigation of quality-of-care gaps. Therefore, registry data are more heterogeneous than in clinical trial databases or research registries that select for specific JIA categories. Populated by pediatric rheumatology centers characterized as innovators and early adopters, with a platform to track quality measures, the network is an ideal setting to test the health equity principles outlined by AHRQ as a research prototype DHT is translated into a viable clinical tool. Qualitative research with anticipated end-users (clinicians and patients) from diverse clinic settings and backgrounds, will increase adaptability. User-centered design expertise in the iterative design and development of the tool, use of QI approaches to pilot the integration of the tool into the clinical workflow are factors that are increasing likelihood of successful future adoption. Barriers to use are anticipated with respect to integration of the DHT into the local EMR interface requiring local leadership buy-in and resources, increasing complexity of real world data to be integrated into updating treatment algorithms, time constraints of introducing new technology and presenting data to patients for shared decision making, required training of clinicians and staff, legal and regulatory requirements related to data flows, potential dependency on technology access and concern for introducing health inequities, need for cross-cultural and language translation in using the tool with languages other than English.

## Discussion

Advances in use of digital technologies, including health information technologies and real-world data analytical technologies, and the increased incorporation of digital devices in our daily lives, create the context and environment for digital CDS tools to be offered at POC with promise to deliver more efficient, effective patient-centered care. The increasing sophistication of EMR and real-world data captured by modern technology into registries creates the opportunity for achieving evidence-based personalized medicine. These data sources together with the appropriate methods, and emerging infrastructures hold much promise to enable patients and physicians to make shared, informed decisions tailored to an individual patient by learning from the experiences of “alike” patients.

However, real-world data can be misleading. Unlike clinical trials, patients are prescribed treatment based on their disease indication (treatment-by-indication), and patients who fail to respond may then be put on an alternative or additional treatment (post-treatment selection bias). Without carefully managing such treatment-by-indication and time-varying post-treatment selection biases using causal inference methods, we cannot obtain unbiased real-world evidence. RCTs are useful for informing population-averaged treatment but are rarely sufficient to inform patient-centered adaptive treatment effect. The causal inference methodologies addressing HTEs and the time-varying adaptive treatment strategy, are increasingly sophisticated and able to handle real world complexity, application of the method requires advanced knowledge and computation programming skill, as well as the ability to harmonize and access multiple data sources.

We envision capability for patient-centered causal learning as the engine of a new kind of smart CDS in form of a DHT. It can utilize large heterogeneous data sources and address multiple data challenges inherent in use of EHR data. The DHT will then bring patient-focused evidence on the comparative effectiveness of treatments in patients like them to the POC and thus improve patient-centered treatment choices. A DHT works well within the scope of a learning health system, which can feed data from clinical care to support shared learning and inform treatment algorithms, leverage QI approaches and a drive towards health equity in development, to test and implement the system equitably in clinical care.

## Data Availability

The data analyzed in this study is subject to the following licenses/restrictions: The datasets analyzed for this study are housed at Cincinnati Children's Hospital Medical Center on a secure server. De-identified data are accessible on reasonable request. Requests to access these datasets should be directed to bin.huang@cchmc.org.
